# Fully automated VMAT treatment planning for advanced-stage NSCLC patients

**DOI:** 10.1007/s00066-017-1121-1

**Published:** 2017-03-17

**Authors:** Giuseppe Della Gala, Maarten L. P. Dirkx, Nienke Hoekstra, Dennie Fransen, Nico Lanconelli, Marjan van de Pol, Ben J. M. Heijmen, Steven F. Petit

**Affiliations:** 1 Department of Radiation Oncology, Erasmus MC Cancer Institute, 5201, 3008 AE Rotterdam, The Netherlands; 2 Scuola di Scienze, Alma Mater Studiorum, Università di Bologna, Bologna, Italy; 3 Department of Radiation Oncology, Massachusetts General Hospital—Harvard Medical School, Boston, MA USA

**Keywords:** Radiotherapy, intensity-modulated, Volumetric-modulated arc therapy, Computer-assisted radiotherapy planning, Non-small cell lung carcinoma, Organs at risk, Intensitätsmodulierte Strahlentherapie, Volumenmodulierte Arc Therapie, Vollautomatisierte Strahlentherapieplanung, Nicht-kleinzelliges Bronchialkarzinom, Risikoorgane

## Abstract

**Purpose:**

To develop a fully automated procedure for multicriterial volumetric modulated arc therapy (VMAT) treatment planning (autoVMAT) for stage III/IV non-small cell lung cancer (NSCLC) patients treated with curative intent.

**Materials and methods:**

After configuring the developed autoVMAT system for NSCLC, autoVMAT plans were compared with manually generated clinically delivered intensity-modulated radiotherapy (IMRT) plans for 41 patients. AutoVMAT plans were also compared to manually generated VMAT plans in the absence of time pressure. For 16 patients with reduced planning target volume (PTV) dose prescription in the clinical IMRT plan (to avoid violation of organs at risk tolerances), the potential for dose escalation with autoVMAT was explored.

**Results:**

Two physicians evaluated 35/41 autoVMAT plans (85%) as clinically acceptable. Compared to the manually generated IMRT plans, autoVMAT plans showed statistically significant improved PTV coverage (V_95% _increased by 1.1% ± 1.1%), higher dose conformity (R_50_ reduced by 12.2% ± 12.7%), and reduced mean lung, heart, and esophagus doses (reductions of 0.9 Gy ± 1.0 Gy, 1.5 Gy ± 1.8 Gy, 3.6 Gy ± 2.8 Gy, respectively, all *p* < 0.001). To render the six remaining autoVMAT plans clinically acceptable, a dosimetrist needed less than 10 min hands-on time for fine-tuning. AutoVMAT plans were also considered equivalent or better than manually optimized VMAT plans. For 6/16 patients, autoVMAT allowed tumor dose escalation of 5–10 Gy.

**Conclusion:**

Clinically deliverable, high-quality autoVMAT plans can be generated fully automatically for the vast majority of advanced-stage NSCLC patients. For a subset of patients, autoVMAT allowed for tumor dose escalation.

## Zusammenfassung

**Zielsetzung:**

Entwicklung einer vollautomatisierten, auf multiplen Kriterien basierenden volumenmodulierten Arc-Therapie-(VMAT-)Behandlungsplanung (autoVMAT) für kurativ behandelte Patienten mit nicht-kleinzelligem Bronchialkarzinom (NSCLC) im Stadium III/IV.

**Material und Methoden:**

Nach Konfiguration unseres autoVMAT-Systems für NSCLC wurde für 41 Patienten der autoVMAT-Plan mit dem manuell erzeugten, klinisch applizierten intensitätsmodulierten Strahlentherapieplan (IMRT) verglichen. AutoVMAT-Pläne wurden ferner mit manuellen und ohne Zeitdruck erstellten VMAT-Plänen verglichen. Für 16 Patienten mit reduzierter Dosisverordnung des Planungszielvolumens (PTV) im klinischen IMRT-Plan (zur Vermeidung einer Verletzung von Toleranzdosen für Risikoorgane) wurde das Potenzial für eine Dosiseskalation mit autoVMAT untersucht.

**Ergebnisse:**

Zwei Ärzte bewerteten 35 von 41 autoVMAT-Plänen (85 %) als klinisch akzeptabel. Verglichen mit den manuell erzeugten IMRT-Plänen zeigten autoVMAT-Pläne eine statistisch signifikant bessere PTV-Abdeckung (V_95%_ erhöht um 1,1 % ± 1,1 %), eine höhere Dosiskonformität (R_50_ verringert um 12,2 % ± 12,7 %) und eine geringere durchschnittliche Dosis in Lunge, Herz und Oesophagus (verringert um je 0,9 Gy ± 1,0 Gy, 1,5 Gy ± 1,8 Gy, 3,6 Gy ± 2,8 Gy; alle *p* < 0,001). Um die restlichen 6 autoVMAT-Pläne aus klinischer Sicht akzeptabel zu machen, benötigte ein Dosimetrist zur Feinabstimmung jeweils weniger als 10 min. AutoVMAT-Pläne wurden verglichen zu manuell optimierten VMAT-Plänen als gleichwertig oder sogar besser erachtet. Für 6 von 16 Patienten ermöglichte autoVMAT eine Dosiseskalation im Tumor um 5–10 Gy.

**Schlussfolgerung:**

Für die große Mehrheit von Patienten mit fortgeschrittenem NSCLC konnte ein klinisch applizierbarer, hochqualitativer autoVMAT-Plan vollautomatisch erstellt werden. Für eine Subgruppe ermöglichte autoVMAT eine Dosiseskalation im Tumor.

In recent years, the use of volumetric modulated arc therapy (VMAT) for treatment of locally advanced non-small cell lung cancer (NSCLC) patients has grown. The main advantage compared to fixed-field intensity-modulated radiotherapy (IMRT) is its improved delivery efficiency. The shorter treatment time of VMAT allows for increased patient throughput, reduced intrafractional motion, and improved patient comfort [1].

IMRT and VMAT treatment planning with commercial treatment planning systems (TPS) is an iterative trial-and-error process. The procedure is time consuming and the quality of the final treatment plan may depend highly on the experience and skills of the dosimetrist, the complexity of the case, and the available time. In addition, there is no guarantee that the final treatment plan reflects the optimal dose distribution for a specific patient in terms of PTV coverage, doses in organs at risk (OAR), and the desired tradeoffs between them.

To increase the consistency and quality of treatment plans, and to reduce treatment planning time, there is a growing interest in automated treatment planning [2–16]. Different vendors offer so-called knowledge-based automated treatment planning solutions. These methods rely on a database of previously treated plans that are used to predict achievable dose objectives for a new patient. For NSCLC patients, several methods for automated treatment planning have been evaluated. Fogliata et al. used Varian’s RapidPlan (Varian Medical Systems, Palo Alto, CA, USA), which applies a database of previously treated plans [12]. Tol et al. used an automated interactive optimization procedure for VMAT plan optimization based on dose–volume constraints and dose–volume histogram analyses [4]. This method requires that the user knows the input priorities that result in acceptable treatment plans for the majority of patients. Zhang et al. and Quan et al. proposed a completely automated process based on in-house developed optimization algorithms [1, 5]. Beam angles were optimized using a library of plans from previously treated patients. For VMAT and IMRT planning techniques, they showed that automatically generated plans were generally preferred by oncologists when compared to manually generated plans. Recently, Kamran et al. investigated the benefit of IMRT planning in combination with multicriterial optimization [13]. Compared to manually generated plans without multicriterial optimization, OAR sparing could be significantly improved without comprising target coverage. Consequently, the multicriterial optimized plans were preferred by the physicians.

In the authors’ department, Erasmus-iCycle was developed, a system for fully automated multicriterial treatment plan generation. Currently, Erasmus-iCycle is clinically applied for IMRT and VMAT plan generation for prostate, head and neck, and cervical cancer patients [3, 6, 14]. The authors have previously demonstrated that automatically generated plans were of noninferior or higher quality compared to plans generated by an expert dosimetrist in the absence of time pressure [3].

The goal of this study was to configure, test, and implement Erasmus-iCycle for automated, multicriterial VMAT treatment planning (autoVMAT) for stage III/IV NSCLC patients treated with curative intent. For 41 consecutively treated patients, autoVMAT plans were compared to manually generated, clinically delivered IMRT plans using dosimetric indices and physicians’ scoring. For a subgroup of patients, autoVMAT plans were also compared to manually generated VMAT plans. Sixteen out of 41 patients had a reduced PTV dose prescription in the clinical plan to avoid violation of OAR tolerance doses. For these patients, the potential for dose escalation with autoVMAT was explored.

## Materials and methods

### Patients and treatment

Two sets of locally advanced stage III–IV NSCLC patients treated with curative intent with IMRT at the Erasmus MC Cancer Institute were included in this study. The first set, consisting of 7 patients treated in 2014, was used to configure the system for automated planning. The second set, consisting of 41 consecutive patients treated between January and August 2015, was used for validation. All clinically delivered IMRT plans consisted of 5–9 beams and were generated with the common manual trial-and-error planning approach, using the Monaco TPS version 5.0 (Elekta AB, Stockholm, Sweden).

For segmentation of the targets and OARs, the 50% exhale phase of a four-dimensional computed tomography (4DCT) scan was used. The clinical target volume (CTV) was defined by expanding the gross tumor volume (GTV) by a margin of 5 mm. If needed, the CTV was manually edited based on anatomic borders. Lymph node stations with affected nodes—as determined on diagnostic CT or positron-emission tomography (PET) scans, endoscopic ultrasound, or fine needle aspiration pathology examination—were also defined as CTV. To define the planning target volume (PTV), the CTVs of the primary tumor and lymph nodes were expanded by a margin of 1 cm in the lateral and ventral–dorsal directions and by a margin of 1–1.2 cm in the cranial–caudal direction, depending on respiratory tumor motion amplitude. According to protocol, the intended PTV dose prescription was 66 Gy. In practice, prescribed doses varied between 30 and 66 Gy, in order to avoid violation of clinical constraints. For 25 patients treated with concurrent chemotherapy, the dose was delivered in fractions of 2 Gy; 15 patients treated with sequential chemotherapy received 2.75 Gy per fraction. The other 8 patients, receiving a prescribed dose of 45 Gy or lower, were treated with 3 Gy per fraction.

Planning goals were to cover at least 95% of the PTV and 99% of the CTV with 95% of the prescribed dose, and 99% of the PTV with 90% of the prescribed dose, while sparing OARs as much as possible. The mean dose in the total lung volume minus GTV had to be kept below 20 Gy and the V_20Gy_ (volume receiving 20 Gy or more) below 35%. Maximum allowed doses to the spinal cord and plexus brachialis were 46.3 Gy and 56.6 Gy (3 Gy/fraction), 49.3 Gy and 60 Gy (2.75 Gy/fraction), and 50 Gy and 66 Gy (2 Gy/fraction), respectively. The protocol also prescribed a V_5Gy_ < 60% for lungs minus GTV and V_45Gy_ < 25% for the esophagus, but these requirements did not determine whether a plan was deemed acceptable or not. A high level of dose conformity was desired.

### Automated multicriterial treatment planning

The in-house developed Erasmus-iCycle TPS cannot be directly used for clinical plan generation. Therefore, automated multicriterial treatment planning was implemented as a two-step process, combing Erasmus-iCycle and the clinically used Monaco TPS. The process is described in detail by Voet et al. [3] and is briefly summarized here. In the first step, Erasmus-iCycle is used to automatically generate an equi-angular 23-beam IMRT plan, simulating VMAT delivery. Further increasing the number of beams increased calculation times but did not lead to improved plan quality [3]. Next, using the achieved constraint and objective values in the Erasmus-iCycle plan, a patient-specific Monaco template is automatically generated, followed by automated plan generation in Monaco based on this template, to result in a clinically deliverable VMAT plan that closely mimics the initial Erasmus-iCycle plan. The same system is used clinically for automated clinical plan generation for prostate, head and neck, and cervical cancer patients [3, 6, 14].

Plan generation with Erasmus-iCycle is based on a site-specific “wishlist”, with hard planning constraints (never to be violated) and *prioritized* objectives. The assigned objective priorities are used to automatically steer the multiobjective plan generation, ensuring clinically desirable tradeoffs between all treatment objectives. Generally, adequate PTV coverage has the highest priority, followed by the most important OAR objective.

In this study, all Erasmus-iCycle plans were generated by employing one fixed wishlist, with some minor variations to handle differences in dose prescription and fractionation (see above). An initial wishlist was composed based on previous clinical experience, the planning protocol, and wishes of treating physicians on how to improve clinically applied plans. It was used to automatically generate a plan for the first 7 patients included in this study. These plans were then evaluated together with physicians, and the wishlist was modified according to their input. This iterative procedure continued until no further improvements in plan quality were achieved for the 7 training patients. The final wishlist is shown in Table 1.

**Table 1:** Erasmus-iCycle wish list for advanced stage NSCLC. The wishlist consists of six hard constraints that are met per definition and 13 objectives that are optimized in order of priority. Once the goal of an objective is achieved or when further optimization is no longer possible, the optimizer fixes the achieved value of the objective (with a bit of slack) as a constraint and continues with the next objective. The first priority was proper CTV and PTV coverage, ensured by the use of the LTCP cost function with a sensitivity value of 0.8 [17]. When the spinal cord was close to the target or overlapped it, the objective was applied to the target volume from which the spinal cord expanded by 5 mm was subtracted. The purpose was to locate the acceptable underdosage to the PTV near the spinal cord. The third objective aims to reduce the maximum dose at 4 cm from the PTV to 60% of the prescribed dose. The next objective tries to reduce the mean lung dose to 15 Gy. Two more objectives (shell PTV + 1 cm and shell PTV + 3 cm) with priorities 5 and 6 realize a steep dose falloff outside the PTV. To minimize the esophagus volume treated to a high dose, an EUD objective were used. Objectives 8 to 13 aimed at further reducing the doses to the OARs. In the final phase of the optimization, all objectives are consecutively minimized to their fullest extent

Priority	Volume	Dose metrics	Limit/goal	Parameter
Constraint	Patient	Max	107% of prescribed dose	–
Constraint	CTV	Max	107% of prescribed dose	–
Constraint	PTV—(spinal cord + 5 mm)	Max	107% of prescribed dose	–
Constraint	Spinal cord + 3 mm	Max	47 Gy	–
Constraint	Plexus	Max	63 Gy	–
Constraint	Lungs—GTV	Mean	19 Gy	–
*1*	CTV	↓ LTCP	1	α = 0.8
*2*	PTV	↓ LTCP	0.5	α = 0.8
*3*	Patient—(PTV + 4 cm)	↓ Max	60% of prescribed dose	–
*4*	Lungs—GTV	↓ Mean	15 Gy	–
*5*	Shell PTV + 1 cm	↓ Max	90% of prescribed dose	–
*6*	Shell PTV + 3 cm	↓ Max	80% of prescribed dose	–
*7*	Esophagus	↓ EUD	44 Gy	k = 8
*8*	Esophagus	↓ Mean	15 Gy	–
*9*	Lungs—GTV	↓ Mean	5 Gy	–
*10*	Heart	↓ Mean	15 Gy	–
*11*	Spinal cord + 3 mm	↓ Max	42 Gy	–
*12*	Unspecified tissue	↓ Max	40 Gy	–
*13*	Plexus	↓ Mean	20 Gy	–

*LTCP* logarithmic tumor control probability,* EUD* equivalent uniform dose,* CTV* clinical target volume, *GTV* gross tumor volume,* PTV* planning target volume, *NSCLC* non-small cell lung cancer, *OAR* organs at risk. The arrows indicate that objectives are minimized

### Comparison of autoVMAT and clinically applied IMRT plans

For the second group of 41 patients, the manually generated IMRT plans were compared with autoVMAT plans using dosimetric indices and by independent scoring by two physicians. For fair comparison, in this part of the investigation, the prescribed tumor dose for the autoVMAT plans was identical to that used in the corresponding clinical plans.

### Comparison of automatically and manually generated VMAT plans

At the time of this study, the authors did not yet apply VMAT in clinical routine for advanced-stage NSCLC patients. Prior to clinical introduction, for 10/41 validation patients the autoVMAT plans were also compared with VMAT plans that were manually generated by an expert dosimetrist without planning time limitations. The dosimetrist had no prior knowledge of the Erasmus-iCycle plans of these patients.

### Dose escalation

In clinical routine, when it was not possible to achieve the intended dose prescription of 66 Gy without violating at least one of the clinical hard constraints, the number of planned fractions, and consequently the prescribed dose, was reduced following a standard schedule: 1. reduction of the prescribed dose to 60 or 60.5 Gy (depending on fractionation schedule), 2. if plan generation was still unfeasible, further reduction to 55 Gy, or, if needed, to 45, 39, or 30 Gy delivered in 3 Gy per fraction. Intermediate dose levels were not clinically applied due to planning time limitations. For the 16 patients with a prescription dose lower than 66 Gy, it was attempted to escalate the dose using autoVMAT, following the standard schedule steps backwards: from 45 Gy to 55 Gy, from 55 Gy to 60.5 Gy, from 60 Gy or 60.5 Gy to 66 Gy. For fair comparison with the clinically applied procedure, intermediate dose escalations were not investigated.

### Statistics

Plan conformity was quantified using R_50_, the ratio between the total volume receiving at least 50% of the prescribed dose and the volume of the PTV. Dose homogeneity in the PTV was measured using the homogeneity index (HI), defined as HI = (D_2%_ − D_98%_)/D_50%_, where D_2%_, D_98%_, and D_50%_ are the doses covering 2, 98, and 50% of the PTV, respectively. Differences in dosimetric indices were expressed as mean ±1 standard deviation. Statistical significance of differences in dosimetric indices was evaluated with two-tailed Wilcoxon signed-rank tests, considering *p* < 0.05 statistically significant.

## Results

### AutoVMAT and clinically applied IMRT plans

**Fig. 1:**
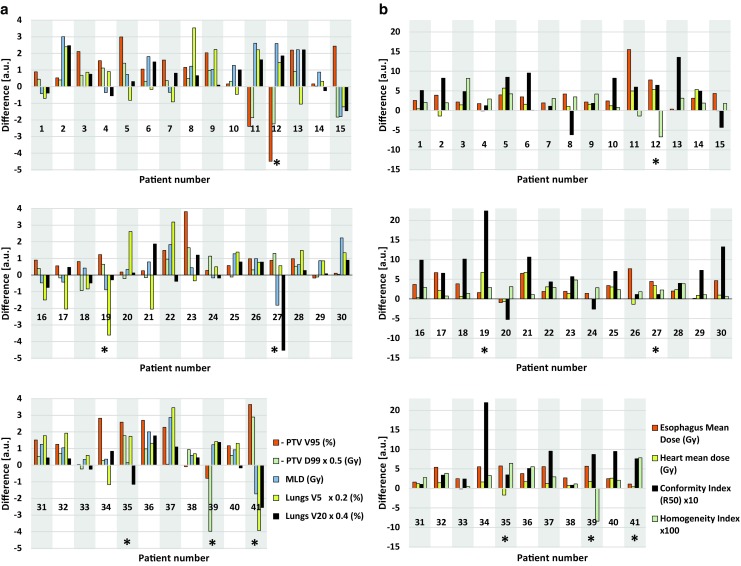
Differences between clinical intensity-modulated radiotherapy (IMRT) and automated volumetric modulated arc therapy (autoVMAT) plans for **a** planning target volume (*PTV*) V_95%_, PTV D_99%_, mean lung dose (*MLD*), lungs V_5Gy_, lungs V_20Gy_; **b** esophagus mean dose, heart mean dose, conformity index (R_50_), and homogeneity index (HI). Positive values are in favor for the autoVMAT plans. V_95%_ is the PTV volume receiving at least 95% of the prescribed dose, D_99%_ is the minimum dose delivered to 99% of the PTV, V_5Gy_ and V_20Gy_ are the volumes receiving 5 Gy, respectively 20 Gy, or more. For visualization purposes, the differences of PTV D_99%_, lungs V_5Gy_ and V_20Gy,_ R_50_, and HI were multiplied by 0.5, 0.2, 0.4, 10, and 100, respectively. Patients for which the autoVMAT plan was rejected are denoted by an *. The pronounced predominance of positive bars confirms the overall improvement in plan quality for autoVMAT plans with respect to the corresponding clinical IMRT plans

Two physicians judged 35/41 of the autoVMAT plans (85%) as clinically acceptable. These plans were considered equivalent or (as in most cases) better than the corresponding IMRT plans, due to a combination of better PTV coverage and dose conformity, as well as sparing of the lungs, heart, and esophagus (Fig. 1 and Table 2). PTV volume receiving at least 95% of the prescribed dose (V_95%_) was on average improved by 1.1% ± 1.1% (*p* < 0.001) and the near-minimum dose in the PTV (D_99%_) by 0.6 Gy ± 1.5 Gy (*p* = 0.004). The mean lung dose (lungs excluding GTV) was reduced by 0.9 Gy ± 1.0 Gy (*p* < 0.001), V_20Gy_ by 1.3% ± 2.1% (*p* = 0.001), and V_5Gy_ by 3.2% ± 7.6% (*p* = 0.023). The esophagus mean dose was on average 3.6 Gy ± 2.8 Gy (*p* < 0.001) lower and V_45Gy_ was reduced by 4.6% ± 4.8% (*p* < 0.001). The heart mean dose was reduced by 1.5 Gy ± 1.8 Gy (*p* < 0.001).

**Table 2:** Mean values and uncertainties, reported as one standard deviation (1 SD), for the evaluated dose metrics of the clinical IMRT plans and the autoVMAT plans for the 35 study patients with acceptable autoVMAT plans. For all metrics, statically significant differences were observed in a two-tailed Wilcoxon signed-rank test

	Clinical IMRT		autoVMAT		*p*-value Wilcoxon test
Dose metrics	Mean	SD	Mean	SD	
PTV V_95% _(%)	97.0	1.4	98.2	1.1	<0.001
PTV D_99%_ (Gy)	55.2	8.9	55.8	9.1	0.004
Conformity index R_50_ (–)	4.67	0.85	4.13	0.56	<0.001
Homogeneity index (–)	0.127	0.035	0.101	0.035	<0.001
Mean lung dose (Gy)	17.3	2.6	16.5	2.3	<0.001
Lungs V_5Gy _(%)	56.0	10.6	52.8	9.7	0.023
Lungs V_20Gy _(%)	29.4	4.8	28.1	4.4	0.001
Mean heart dose (Gy)	12.3	6.6	10.8	5.9	<0.001
Mean esophagus dose (Gy)	24.4	6.9	20.8	6.5	<0.001
Esophagus V_45Gy_ (%)	27.0	13.2	22.4	11.9	<0.001

*IMRT* intensity-modulated radiotherapy, *autoVMAT* automated volumetric modulated arc therapy, *PTV* planning target volume. V_95%_ is the PTV volume receiving at least 95% of the prescribed dose, D_99%_ is the minimum dose delivered to 99% of the PTV. V_5Gy_, V_20Gy_ and V_45Gy_ are the volumes receiving respecively 5 Gy, 20 Gy and 45 Gy, or more

The autoVMAT plans were generally more conformal than the clinical IMRT plans as indicated by a reduction in R_50_ by, on average, 12.2% ± 12.7% (*p* < 0.001). Moreover, the PTV dose homogeneity improved by 0.025 ± 0.017 (*p* < 0.001), i. e., an average reduction of 20%.

### Unacceptable plans

For 6 patients, the physicians deemed the autoVMAT plan clinically unacceptable due to violation of at least one clinical constraint. In 2 patients PTV coverage was not adequate (V_95_ < 95%, V_90_ < 99%); for 4 patients the MLD and/or lungs V_20Gy_ exceeded clinical constraints. It took a dosimetrist less than 10 min hands-on time to manually fine-tune these plans in Monaco, rendering them acceptable. In contrast, 3–4 h were required to generate a VMAT plan from scratch.

### AutoVMAT and manually generated VMAT plans

**Fig. 2:**
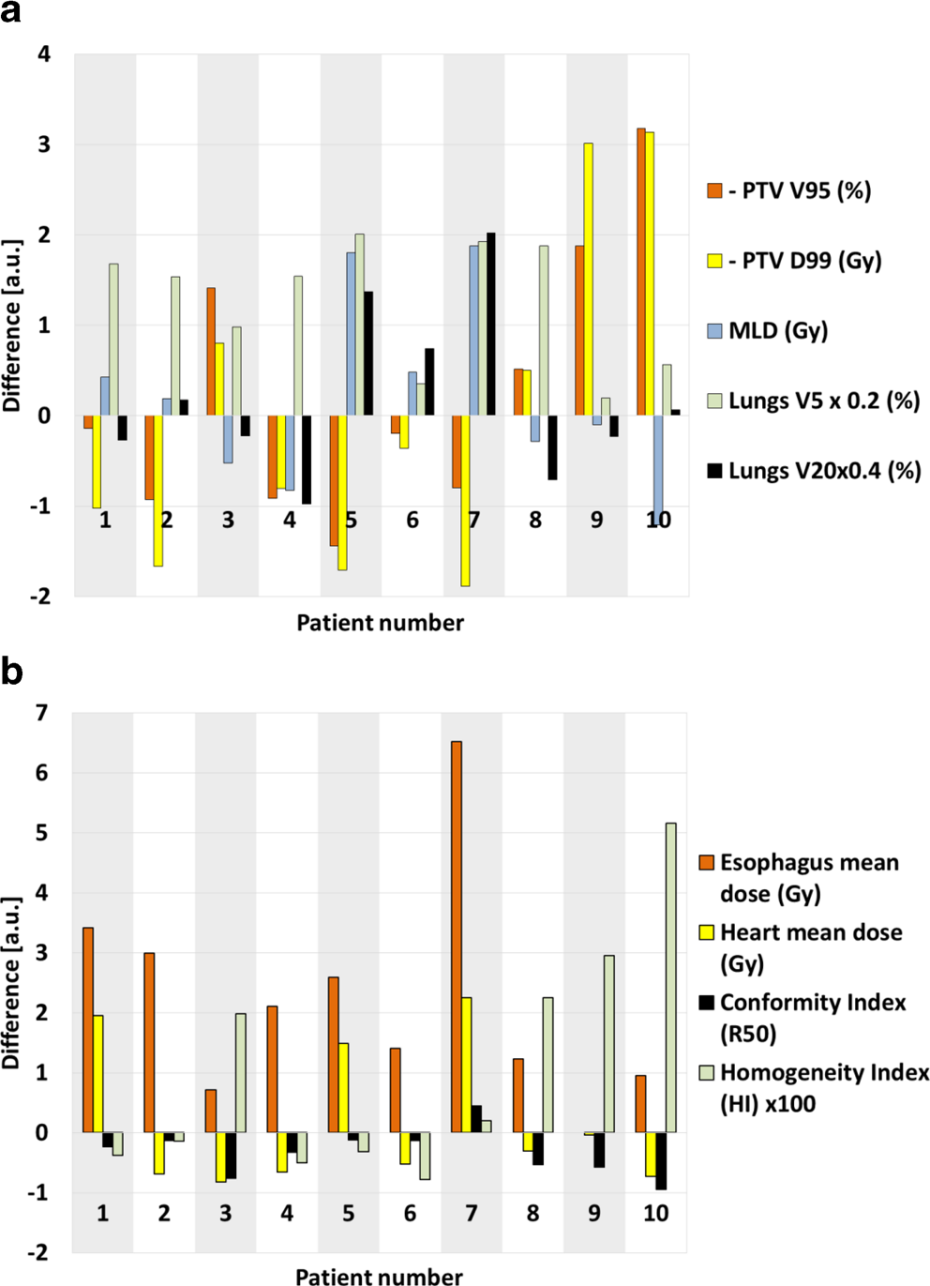
Differences between manually generated volumetric modulated arc therapy (VMAT) plans and automated VMAT (autoVMAT) plans for **a** planning target volume (*PTV*) V_95%, _PTV D_99%,_ mean lung dose (*MLD*), lungs V_5Gy_, and lungs V_20Gy_, and **b** esophagus mean dose, heart mean dose, conformity index (R_50_) and homogeneity index (HI). V_95%_ is the PTV volume receiving at least 95% of the prescribed dose, D_99%_ is the minimum dose delivered to 99% of the PTV, V_5Gy_ and V_20Gy_ are the volumes receiving 5 Gy, respectively 20 Gy, or more. Positive values are in favor of the autoVMAT plans. For visualization purposes, the differences of the lungs V_5Gy_ and V_20Gy_ and HI were multiplied by 0.2, 0.4, and 100, respectively. Positive values for PTV coverage are often coupled with negative values for MLD and vice versa

A radiation oncologist evaluated all autoVMAT plans as equivalent or better in quality compared to the manually generated VMAT plans. Average differences were not statistically significant, except for lungs V_5Gy_ (reduction by 6.3% ± 3.3%; *p* = 0.008), esophagus mean dose (reduction by 2.2 ± 1.8 Gy; *p* = 0.012), and esophagus V_45Gy_ (reduction by 3.2% ± 2.3%; *p* = 0.012), all in favor of autoVMAT plans. The manually generated plans were slightly more conformal, as expressed by a difference in R_50_ of 8% ± 10% (*p* = 0.038). The differences in dose metrics are shown in Fig. 2.

### Dose escalation

For 6/16 patients with a prescription dose less than 66 Gy, a clinically acceptable autoVMAT plan with a higher prescribed tumor dose could be generated. In 2 patients dose escalation from 45 to 55 Gy was possible, in 2 further patients from 60 to 66 Gy. For 1 patient the dose could be escalated from 55 to 60.5 Gy and for another from 60.5 to 66 Gy. In all cases, the physicians preferred the plan with the higher tumor dose.

## Discussion

This study developed a method for fully automated VMAT treatment planning for NSCLC patients based on Erasmus-iCycle. A single wishlist was used for all patients treated with the same protocol. The Erasmus-iCycle dose distributions were converted automatically into clinically deliverable Monaco VMAT plans. It was shown that autoVMAT plans were of equivalent or, as in most cases, higher quality than the clinically applied IMRT plans. This may be attributed partly to the delivery technique (VMAT vs. IMRT), as was also observed previously [1], and partly to the difficulty of manually selecting the optimal IMRT beam configuration for individual patients. AutoVMAT plans were also of a higher or equivalent quality when compared to VMAT plans generated by an expert dosimetrist in the absence of time constraints. Only in 6/41 cases were autoVMAT plans not clinically acceptable, and a dosimetrist needed only 10 min to make these plans fulfil the constraints. The automated procedure led to a decrease in hands-on treatment planning time of 3–4 h per patient compared to conventional VMAT planning. Based on these results, autoVMAT has been clinically applied for all advanced-stage NSCLC patients treated with curative intent at the authors’ hospital since March 2016.

In previous studies for prostate, head and neck, and cervical cancer [3, 6, 14], it was not observed that some of the autoVMAT plans were unacceptable and of a lesser quality than manually generated IMRT plans with the same prescribed dose. The authors believe that this may be attributed to the simple pencil beam dose calculation algorithm in Erasmus-iCycle [17]. For NSCLC patients, this may result in high-quality Erasmus-iCycle plans that cannot be accurately mimicked with the advanced Monte Carlo dose engine in Monaco. Therefore, in an ongoing project, the Monte Carlo engine used in Monaco is coupled to Erasmus-iCycle. It is expected that, in the near future, this will avoid generation of unacceptable plans for NSCLC patients as well.

For a considerable proportion of advanced-stage NSCLC patients, a prescribed dose of 66 Gy is not achievable. In these cases, the prescribed dose has to be lowered to yield acceptable OAR doses. This can be a tedious process and the clinical protocol was therefore to lower the prescribed dose in fixed steps of multiple fractions. With autoVMAT it is now feasible to apply individualized dose reductions, avoiding PTV doses lower than strictly needed.

Erasmus-iCycle for autoVMAT planning has a couple of advantages compared to other approaches for automated (VMAT) planning. First, it does not fully rely on a database of prior patients, as published knowledge-based approaches do [1, 4, 5, 12]. A small set of training patients (≤10) is only used as starting point for establishing a site-specific wishlist. When generating this wishlist, the explicit aim is to achieve a higher plan quality than in the clinical plans of the training patients. With this procedure, automatically generated plans for previously treated patients have, on average, a higher or at least non-inferior plan quality than the clinical plans [3], as also shown in this work. The need for a small set of training patients has an additional advantage in case of changes in clinical protocol, which would otherwise require a new large database of high-quality plans.

## Conclusion

Fully automated VMAT treatment planning was implemented for advanced-stage NSCLC. For the vast majority of patients, autoVMAT plans were equivalent or better than manually generated plans. Moreover, for a subset of patients, autoVMAT allowed for tumor dose escalation within clinical constraints. Consequently, autoVMAT has been applied for NSCLC in routine clinical use at the authors’ hospital since March 2016.

## References

[1] Quan EM, Chang JY, Liao Z (2012). Automated volumetric modulated arc therapy treatment planning for stage III lung cancer: how does it compare with intensity-modulated radio therapy?. Int J Radiat Oncol Biol Phys.

[2] Bezjak A, Rumble RB, Rodrigues G, Hope A, Warde P, Members of the IIEP (2012). Intensity-modulated radiotherapy in the treatment of lung cancer. Clin Oncol (R Coll Radiol).

[3] Voet PW, Dirkx ML, Breedveld S, Al-Mamgani A, Incrocci L, Heijmen BJ (2014). Fully automated volumetric modulated arc therapy plan generation for prostate cancer patients. Int J Radiat Oncol Biol Phys.

[4] Tol JP, Dahele M, Delaney AR, Doornaert P, Slotman BJ, Verbakel WF (2016). Detailed evaluation of an automated approach to interactive optimization for volumetric modulated arc therapy plans. Med Phys.

[5] Zhang X, Li X, Quan EM, Pan X, Li Y (2011). A methodology for automatic intensity-modulated radiation treatment planning for lung cancer. Phys Med Biol.

[6] Voet PW, Dirkx ML, Breedveld S, Fransen D, Levendag PC, Heijmen BJ (2013). Toward fully automated multicriterial plan generation: a prospective clinical study. Int J Radiat Oncol Biol Phys.

[7] Amit G, Purdie TG (2015). Automated planning of breast radiotherapy using cone beam CT imaging. Med Phys.

[8] Sharpe MB, Moore KL, Orton CG (2014). Point/Counterpoint: Within the next ten years treatment planning will become fully automated without the need for human intervention. Med Phys.

[9] Wu B, McNutt T, Zahurak M (2012). Fully automated simultaneous integrated boosted-intensity modulated radiation therapy treatment planning is feasible for head-and-neck cancer: a prospective clinical study. Int J Radiat Oncol Biol Phys.

[10] Fiege J, McCurdy B, Potrebko P, Champion H, Cull A (2011). PARETO: A novel evolutionary optimization approach to multiobjective IMRT planning. Med Phys.

[11] Boylan C, Rowbottom C (2014). A bias-free, automated planning tool for technique comparison in radiotherapy – application to nasopharyngeal carcinoma treatments. J Appl Clin Med Phys.

[12] Fogliata A, Belosi F, Clivio A (2014). On the pre-clinical validation of a commercial model-based optimisation engine: application to volumetric modulated arc therapy for patients with lung or prostate cancer. Radiother Oncol.

[13] Kamran S, Mueller BS, Paetzold P (2016). Multi-criteria optimization achieves superior normal tissue sparing in a planning study of intensity-modulated radiation therapy for RTOG 1308-eligible non-small cell lung cancer patients. Radiother Oncol.

[14] Sharfo AW, Voet PW, Breedveld S, Mens JW, Hoogeman MS, Heijmen BJ (2015). Comparison of VMAT and IMRT strategies for cervical cancer patients using automated planning. Radiother Oncol.

[15] Krayenbuehl J, Norton I, Studer G, Guckenberger M (2015). Evaluation of an automated knowledge based treatment planning system for head and neck. Radiat Oncol.

[16] Hazell I, Bzdusek K, Kumar P (2016). Automatic planning of head and neck treatment plans. J Appl Clin Med Phys.

[17] Breedveld S, Storchi PR, Voet PW, Heijmen BJ (2012). iCycle: Integrated, multicriterial beam angle, and profile optimization for generation of coplanar and noncoplanar IMRT plans. Med Phys.

